# Manuel des Injections Sous-Cutanées

**Published:** 1886-10

**Authors:** 


Manuel des Injections Sous-Cutanees. *Par* Bourneville, *Médécin de Bicêtre, et* Bricon, *Docteur en Médecine. 2me Edition, revue et augmentée. Duodecimo, pp. xl, 200. Paris.*

The second edition of this work is a proof that the method of treating by hypodermic injection is becoming quite common, at least in Europe, whether due to success or to the good nature of Europeans in giving themselves for experimental purposes.

Hypodermic injections being not free from danger of inflammation and abscesses in many cases, it would not be well for a beginner or inexperienced physician to use many of the drugs which are only on trial yet, and from which the results are variable, and often dangerous.

Attention is called to the new drugs and the best vehicle for each one; also how to make hypodermic injection in order to avoid accidents; the care of the syringe, etc.

The hypodermic method may commend itself to the physician who is fond of experiments; but it may not be found equally so for the patient who is sick from a disease which requires frequent administration of medicine hypodermically.

The reader is left to conjectures, as the author does not state whether he believes that the hypodermic method will be the one *par excellence*, or only to be resorted to when it is impossible to introduce medicines into the system through the stomach. Aside from this the book is a good one and well written. D. D.

